# Spatial variance-mass allometry of population density in felids from camera-trapping studies worldwide

**DOI:** 10.1038/s41598-020-71725-0

**Published:** 2020-09-09

**Authors:** Stefano Anile, Sébastien Devillard

**Affiliations:** 1 Cooperative Wildlife Research Laboratory, Southern Illinois University, Carbondale, IL 62901 USA; 2 Laboratoire de Biométrie Et Biologie Evolutive, Univ Lyon, Université Claude Bernard Lyon 1, CNRS, 69100 Villeurbanne, France

**Keywords:** Ecology, Evolution, Zoology, Ecology

## Abstract

Power laws are cornerstone relationships in ecology and evolutionary biology. The density-mass allometry (DMA), which predicts an allometric scaling of population abundance, and Taylor’s law (TL), which predicts a decrease in the population abundance variation along with a decrease in population density, have enhanced our knowledge of inter- and intra-specific variation in population abundance. When combined, these two power laws led to the variance-mass allometry (VMA), which states that larger species have lower spatial variation in population density than smaller species. The VMA has been predicted through theoretical models, however few studies have investigated if this law is also supported by empirical data. Here, to formally test the VMA, we have used the population density estimates obtained through worldwide camera trapping studies for an emblematic and ecologically important carnivorous taxa, the *Felidae* family. Our results showed that the VMA law hold in felids, as well as the TL and the DMA laws; bigger cat species showed less variation for the population density than smaller species. These results have important implications for the conservation of wildlife population and confirm the validity of important ecological concepts, like the allometric scaling of population growth rate and the slow-fast continuum of life history strategies.

## Introduction

Investigating whether ecological laws (sensu biological rules) are supported by empirical data at the macro-ecological scale is a cornerstone in ecology and evolutionary biology^1^. Among ecological laws, power laws have received lots of attention recently, both at the theoretical and empirical levels^1,2^. In general, power law relationships relate a trait, at the individual, population or community level, to another one with a power function Y = βX^α^, therefore leading to a linear relationship at the log-scale^1^; these slopes (i.e. coefficients) are then used to inform on the macro-ecological processes acting on these levels. Power laws have greatly contributed to our understanding of the large-scale variations of population abundance^3–5^ or of variation of metabolism across the body mass continuum in animals and plants^6–8^. In this context, one of the first power law ascertained in ecology is the Taylor's law (hereafter, TL), which positively links the variance in population density to the mean density of populations^3,9^ with the following equation:

1 $$Var_{Di} = \, a \cdot \left( {Mean_{Di} } \right)^{b} ,{\text{ with}}\;a > 0,{\text{ and}},{ 1} \le b \le {2 }\;{\text{in }}\;{\text{numerous }}\;{\text{empirical }}\;{\text{examples}}\; {\text{where}}\;Di\;{\text{is the population density in study site}}\;i.$$

Since the seminal paper of Taylor^3^, this relationship has upheld across numerous taxa^1,9–13^, and has been the subject of several theoretical works^14–16^ aiming to unravel the biological meaning of its exponent (i.e. the slope or coefficient).

Furthermore, another ecological law, the Density-Mass Allometry (hereafter DMA^4,17–20^), relates mean population density to body mass, where density decreases with increasing body mass according to the theory of energetic requirements of species (Eq. 2 below):

2 $${\text{Mean}}_{{{\text{Di}}}} = c \cdot \left( {{\text{Mean}}_{{{\text{BM}}}} } \right)^{d} ,c > 0,{\text{ and}},d < 0,{\text{ where}}\;BM\;{\text{is}}\;{\text{ the }}\;{\text{species }}\;{\text{body }}\;{\text{mass}}$$

Similarly to the TL, also the DMA has been thoroughly researched and results have supported its predictions^4,17,21–33^.

More recently a third ecological law, named the Variance-Mass Allometry (hereafter VMA) combines the two above mentioned laws into one equation (Eq. 3), which links the variance of population density over study sites to the body mass of the species^1,34^,

3 $${\text{Var}}_{{{\text{Di}}}} = ac^{b} .\left( {{\text{Mean}}_{{{\text{BM}}}} } \right)^{bd}$$

that leads a linear relationship at the log10 scale

4 $${\log}\left( {{\text{Var}}_{{{\text{Di}}}} } \right) = {\text{b}}_{0} + {\text{b}}_{1} \cdot {\log}\left( {{\text{Mean}}_{{{\text{BM}}}} } \right),{\text{ with}}\;{\text{b}}_{1} \;{\text{being}}\;\;{\text{ negative}}.$$

Given that both TL and DMA hold in some taxa, it is indeed reasonable to expect that the variance of population density is negatively related to the species body mass (i.e. the bigger the species, the less variation in mean population density). The VMA scaling exponent has been theoretically predicted by combining the TL scaling exponent (∼ 2) and the DMA exponent (∼ − 0.75), giving an exponent for the VMA of ∼ − 3/2^1,34^.

Finding a more general expression for the VMA, without the mandatory step of testing the TL and DMA laws, would help understand the ecological processes which affect variability in population density. A recent study has indeed demonstrated that the VMA should hold even without needing to first test whether the TL and DMA separately hold^35^. Indeed, the model developed by Segura and Perera^35^ assumes that metabolic requirements constrain the maximum abundance of a dominant species in a local community^36–40^ and hence this model suggested the existence of a general form of variance-mass allometry which, under some particular circumstances, includes the VMA previously developed by Marquet^1^ and Cohen et al.^34^. Specifically, Segura and Perera^35^ argued that the explicit link between mean population density and the metabolic scaling can vary due to periodic changes in resources or temperatures and that is why free-living, free-living infested, and parasitic species exhibit different VMA relationships^41^.

Since its first formalization by Cohen et al.^34^, the VMA has been ascertained only through empirical data sets^34,41,42^, but to the best of our knowledge, an empirical test in terrestrial animals is lacking. For example, Cohen et al.^34^ found a strong support for the VMA prediction within genera of oak (*Quercus* sp.) trees; moreover, these authors suggested that the VMA should be evaluated at higher taxonomic levels because both the TL and DMA equations can also be applied among distantly related species^43,44^. Similarly, the results of Xu^42^ support the existence of VMA in oak trees, while also suggesting that the VMA should be true also for fishes. Lagrue et al.^41^ further provided cross-species evidence that the VMA law occurs in a wide range of metazoan parasites, host species, and free-living species without parasites.

Given the aforementioned evidence, it can thus be predicted that population densities of smaller species should be more variable spatially, and likely temporally, than densities of larger-bodied species.

To test the VMA on terrestrial animals, repeated robust estimates of population density across study sites for a representative set of species within a taxon are needed. Clearly, density estimates must be robust, standardized and independent from each other. Camera trap monitoring^45^ has been used extensively to estimate population density or other population parameters of many species of *Felidae*^46,47^. Many cat species are naturally individually marked as their fur-coats feature stripes, rosettes and spots. Hence, with the advent of camera-trapping as a “standard” sampling method, researchers can obtain species-specific density estimates for a variety of felids, making felids an ideal taxon for empirically testing the VMA ecological law.

Terrestrial carnivores are key species which exerted crucial effects not only on the abundance, richness and diversity of the community of species present in a given area, but they are also essential for shaping, regulating and maintaining entire ecosystems^48–50^. Among terrestrial carnivores, the *Felidae* family is a taxa which has capitalized the researcher’s attention since numerous decades, not only because of their charismatic nature^51^ and high conservation value as umbrella species^52^, but also because the largest species (e.g. tiger *Panthera tigris*, lion *Panthera lion*, and leopard *Panthera pardus*) can be, in certain circumstances, man-eaters^53^. Moreover, all the members of the *Felidae* family are obliged carnivores^54^, hence making them particularly prone to the predation of livestock^53^, which in turn can cause severe human-conflicts with retaliatory consequences^53^. Despite this strong research interest in felids^54^, it is still difficult to study them in the wild given their low population densities^55^, their elusive and nocturnal behaviors^56^ as well as the logistic constraints due to the nature of their habitats (e.g. tropical forests, Savannah steppe, high altitude mountains, deserts and Siberian taiga).

Important evolutionary and conservation questions remains to address for this taxa. For example, it is largely unknown how population density in cat species varies spatially or temporally and which factors can trigger these variations. Carnivore density or distribution is generally positively related to prey abundance^57–59^, however such studies have included only one large cat species (i.e. tigers, leopards, and *Panthera onca* jaguars), hence inferences at the family level cannot be generalized. Moreover, testing broader ecological laws regarding population density across *Felidae* might assist biologists seeking to conserve this important taxa.

Our primary goal was to investigate whether the VMA law is supported in wild populations of felids using density estimates collected through camera trap surveys worldwide. Following Cohen et al.^34^, before testing the VMA in felids, we first ascertained whether the TL and the DMA laws were supported,. Given the expected universal aspect of TL, DMA and VMA, we predicted both ecological laws would be supported in felids.

## Results

### Data availability

**Figure 1:**
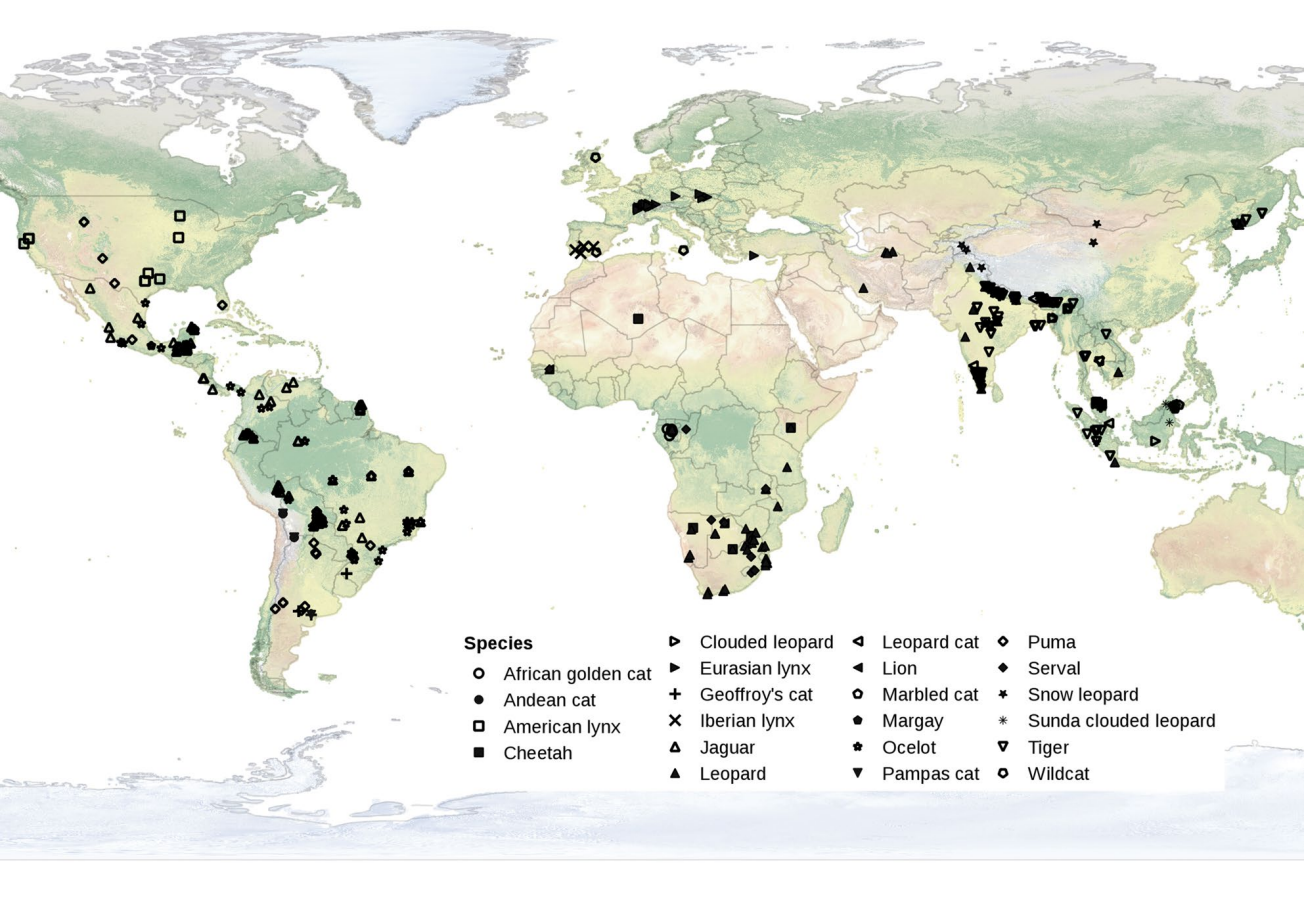
Worldmap of the locations of the *n* = 679 records of population density coming from camera trap studies in 22 Felid species. Only records using CR FMMDM, CR HMMDM and SECR methods of density estimation are shown. The map was generated using Qgis version 1.7 https://qgis.org/en/site/about/index.html.

The literature search achieved an initial dataset of 679 estimates of population density in felids coming from 260 camera trap studies from 54 countries (Fig. 1, Supplementary Information Table S1). Among the 40 recognized felids species^60^), we obtained density estimates from 22 (mean number of records per species = 30.86 ± 45.66 (s.e.m); range [1, 159]). Tiger (*n* = 159), leopard (*n* = 133) and jaguar (*n* = 101) represented the most-represented species, while lion and margay (*Leopardus wiedii*) had only two records each. Twenty-six, 161, and 492 density estimates were from CR FMMDM, CR HMMDM, and SECR methods.

To prepare the final dataset, we first discarded 90 density estimates that were a result of non-targeted, i.e. random, surveys. We then identified the density records which were outside the distribution of density estimates for each pair species/method of density estimation (i.e. density estimates that might be biased due to methodological weaknesses) using a boxplot (Supplementary Information Fig. S1) and 38 additional records were discarded from the dataset. All outliers were unexpectedly high density estimates (Supplementary Information Fig. S1).

The reduced dataset included thus *n* = 551 records for the 22 Felid species (mean = 25.05 ± 39.54 range [1, 142]). From this dataset, we calculated *MeanDensity* and *VarDensity* over specific study sites for each pair (species/method of density estimation). We obtained 27 measures of *MeanDensity* and *VarDensity* calculated over at least three different study sites and for the three methods of density estimation (2 CR FMMDM, 8 CR HMMDM, and 17 SECR) for 18 species. Two species had estimates for three methods of density estimation (Supplementary Information Table S2), 5 for two, and eleven for one. The mean number of specific study site per species/method of density estimation pair was 12.63 ± 14.16 (s.e.m) and ranged from 3 to 57. This final dataset was then used for the following data analysis.

### Investigating TL, DMA and VMA in felids

**Figure 2:**
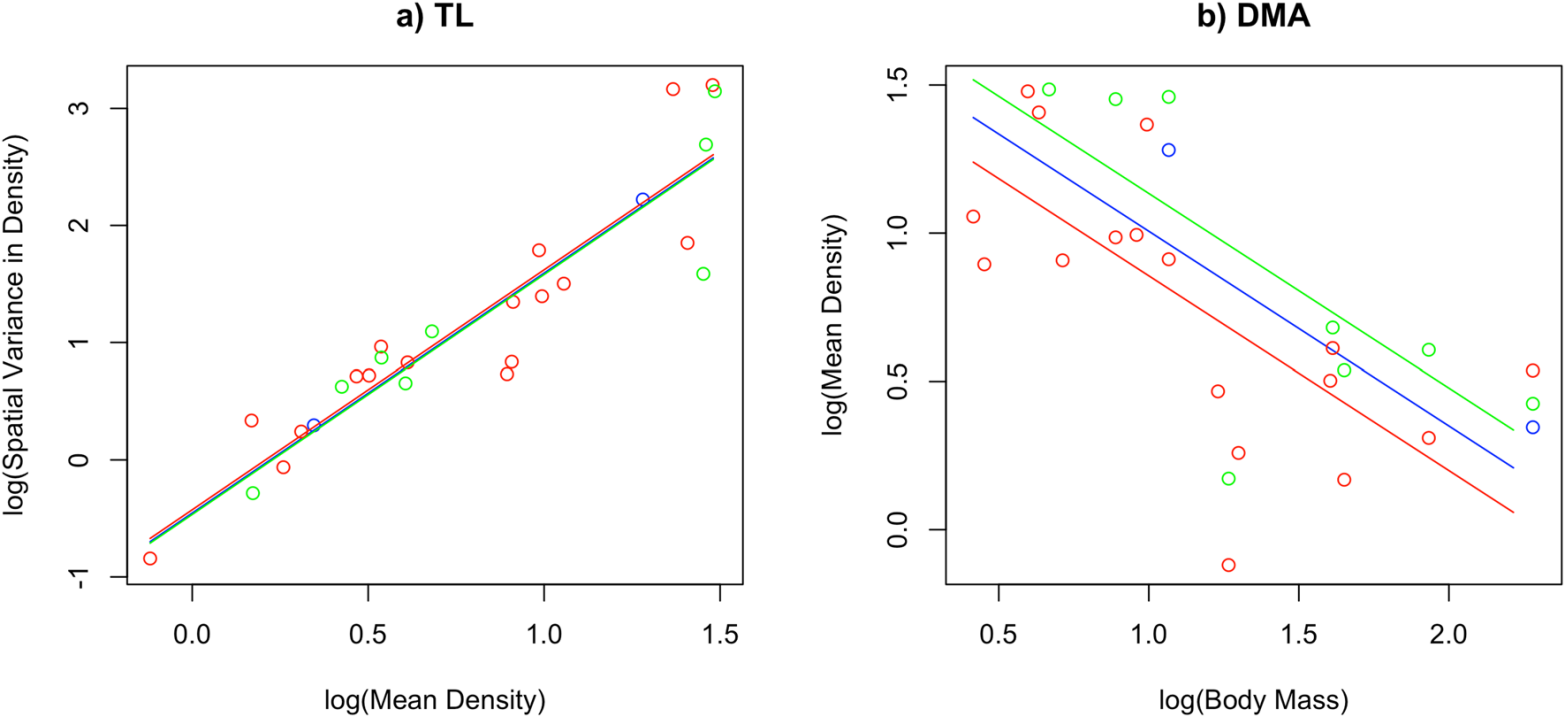
Taylor’s law (**a**) (TL) and Density-Mass Allometry (**b**) (DMA) in 18 species of felids. Predicted value were computed on fixed effects only and from the additive models. Blue points and lines are for the CR FMMDM method of density estimation, green points and lines are for the CR HMMDM method of density estimation, and red points and lines are for the SECR method of density estimation.

The slopes of the TL was not impacted by the method of density estimation as the interaction term DensityMethod*log.MeanDensity was not retained in the model (*p* = 0.9720). Therefore we computed the additive model which showed an effect of l*og.MeanDensit*y on *log.VarDensity* (*p* = 6.10 10^–5^, conditional R^2^ = 0.87) and found no effect of the method of density estimation (*p* = 0.6265). We thus estimated the slope of the TL using the *b* estimates of the additive model: the slope was positive and equal to *β*_log.MeanDensity_ = 2.0470 ± 0.1858 (s.e.m) (*p* = 6.10 × 10^–5^). Therefore the more the mean density over different study sites is, the more the spatial variance in density increases, hence we found strong support to the TL in *Felidae* (Fig. 2a).

The DMA predicted the mean density of species was negatively related to their body mass at the log-scale. Again the slope of the DMA was not significantly different for the three methods of density estimation (interaction term DensityMethod*log.BodyMass p = 0.053). We thus fitted the additive model, and, in this model, the method of density estimation was not significant (p = 0.1266) while the effect of the *log.BodyMass* was highly significant (p = 0.0005, conditional R^2^ = 0.90). DMA was thus strongly supported in Felids (*p* = 0.0005) and the slope of the DMA was to *β*_og.BodyMass_ = − 0.6561 ± 0.1531 (s.e.m) (*p* = 0.0005, Fig. 2b) so the mean density was negatively correlated with their body mass.

**Figure 3:**
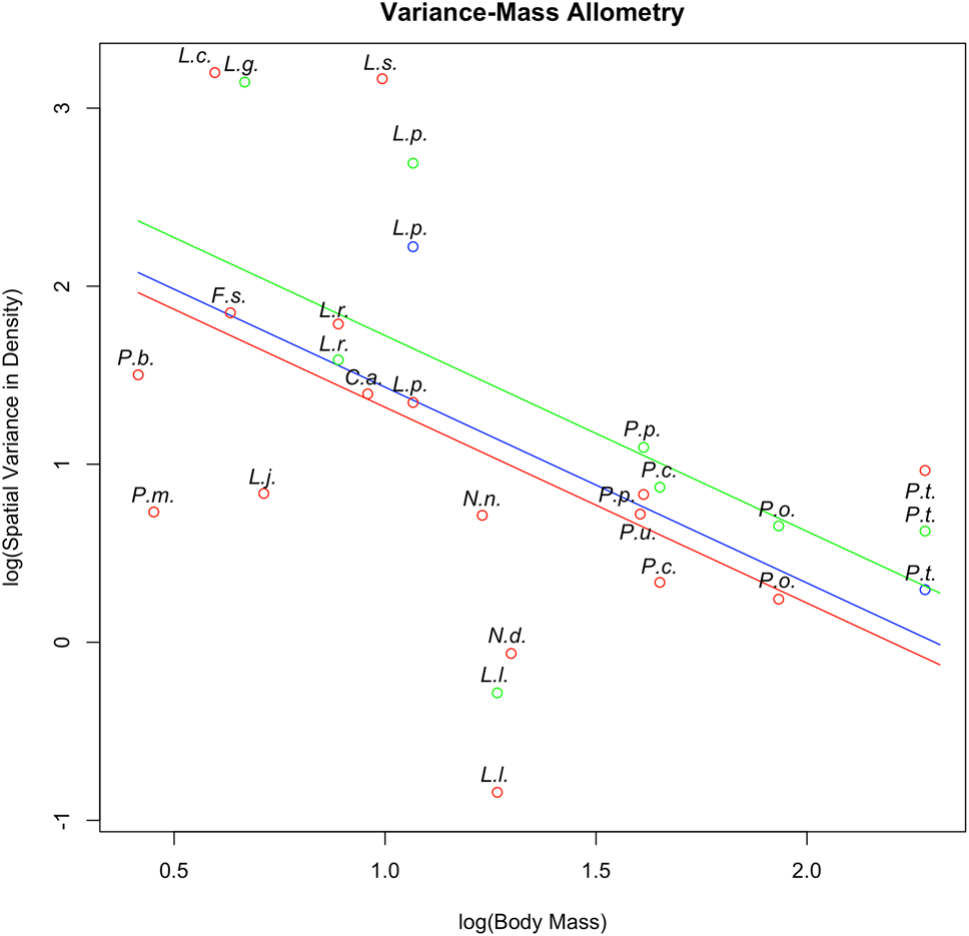
Variance-Mass Allometry (VMA) in 18 species of felids. Predicted values were computed on fixed effects only and from the additive models. Blue points and lines are for the CR FMMDM method of density estimation, green points and lines are for the CR HMMDM method of density estimation, and red points and lines are for the SECR method of density estimation. Species are shown on the plot using their Genus-Specie initials: *C.a.* *Caracal aurata*, *F.s.* *Felis silvestris*, *L.c.* *Leopardus colocolo*, *L.g*. *Leopardus geoffroyi*, *L.j.* *Leopardus jacobita*, *L.p.* *Leopardus pardalis*, *L.s.* *Leptailurus serval*, *L.l.* *Lynx lynx*, *L.r.* *Lynx rufus*, *N.d.* *Neofelis diardi*, *N.n*. *Neofelis nebulosa*, *P.o.* *Panthera onca*, *P.p.* *Panthera pardus*, *P.t.* *Panthera tigris*, *P.u.* *Panthera uncia*, *P.m.* *Pardofelis marmorata*, *P.b.* *Prionailurus bengalensis*, *P.c.* *Puma concolor.*

The interaction between *DensityMethod* and *log.BodyMass* was not significant (*p* = 0.3571), highlighting that the VMA slope is equal which ever the method of density estimation used. No significant effect of the method of density estimation was shown (*p* = 0.3344) while *log.BodyMass* had a significant effect on *log.VarDensity* (p = 0.0207, conditional R^2^ = 0.88) in this additive model. As expected, the VMA slope was negative and equal to *β*_log.BodyMass_ = − 1.099 ± 0.4264 (s.e.m) (*p* = 0.0207, Fig. 3). We found thus that the spatial variance in density for felid species is negatively related to their body mass.

## Discussion

Based on an exhaustive review of population density estimates over multiple study sites from camera-trap studies on felids worldwide, we demonstrated that the spatial VMA was supported for *Felidae*, and hence variation in population density decreases with increasing body mass. We thus provide the first evidence that the spatial variance of population density is a power-law function of average body mass at the interspecific level for terrestrial animals. The VMA was already found for another population parameter across mammalian species as Sinclair^61^ reported an inverse allometric relationship between the standard deviation of the maximum population growth rate and body mass. This and our findings underlined that the general variance-mass allometry recently predicted by the model of Segura and Perera^35^ might hold also for numerous population demographic traits (e.g. survival and reproduction rate, population density, population growth rate) in terrestrial animals. Population density databases for other terrestrial animal taxa are now available^62^, allowing further tests on the spatial density VMA; a more thoroughly understanding of the processes and implications of such universal law would be beneficial, especially when considering the current species extinction crisis^63,64^.

The VMA slope estimated on felids (β_*log.BodyMass*_ = − 1.099) is slightly lower than both the theoretical value of ∼ − 3/2^1,34^ and the expected value of 2.0470 × (− 0.6561) = − 1.343 (Eq. 3) given the estimated slopes of the TL and the DMA. *β*_*log.BodyMass*_ also fell in the lower bound of previous estimated slopes [− 0.29–3.28]^34,41,42^. Differences in lifestyle, metabolic ecology^35^ and resource use among trees^34^, parasites^41^, fishes^42^ and terrestrial carnivores (this study) are likely to explain these discrepancies but it points to a deeper assessment when more VMA slopes are published. Several non-mutual scenarios can explain the difference between the theoretical (− 3/2) and the expected (− 1.343) slope and, alternately, the actual slope of the VMA we found (− 1.099). Different arguments could indeed be formulated to explain this flattening of the VMA encompassing both a lower than expected variability in small species and larger variability than expected in large species. In a more optimistic scenario, our dataset may not be representative enough of the actual variability found in population densities of small cats; a scenario which we consider realistic given the strong bias in research efforts towards large cats^46,47^. In the worst case scenario, we cannot exclude that population densities of small cat populations are truly depressed, hence they are not able to attain their highest predicted population densities, thereby lowering the density variation in small species and ultimately flattening the VMA. For large cat species, the density variation might be higher than expected, thus flattening the VMA. Particularly high or low values for large cat population densities may be either highly biased, as the outliers we discarded from our dataset. High density values can also be genuine strongholds for the conservation of a particular species highlighting efficiency in conservation measures locally while low value might underpin highly endangered populations calling for rapid protection measure locally.

Beyond reporting further evidence of well-known power laws in ecological systems (TL and DMA), our finding on the existence of spatial VMA across felid species has strong implications for the management and conservation of these charismatic species. Many conservation programs aim at increasing population abundance above a minimum viable population density^65^. However, given that larger cat species had less spatially variable population density, it is likely that larger cats are also less prone to respond to conservation actions specifically tailored to increase population density than smaller cats. Therefore, biologists should take into account our results when planning conservation actions involving quantitative objectives on population density. For example, increasing population density by 10% might be far more difficult for a large species than a small one.

How this general pattern relates to the life history of each cat species and its relative conservation status would be a key research topic for future studies. Nonetheless, one might argue that the existence of the VMA law laid in the well-known concept of the slow-fast continuum^66^, which states that larger and slower species displayed higher adult survival, lower annual fecundity and population turnover, so that spatial and temporal variation in abundance is reduced. However, when considering the wide range of body sizes and life histories observed in carnivores, this presumed fast-slow continuum is not well supported^67^. Moreover, also the well-known allometry of the intrinsic rate of increase *r*_*max*_ which scales to body mass with an allometric exponent close to -1/4^68,69^ can also be related to the VMA pattern we observed. As *r*_*max*_ is ultimately controlled by levels of birth, growth and mortality, the allometric scaling of *r*_*max*_ underlines that large species have, among other traits, a slower population’s capability of recovering after population collapses due to numerous types of disturbances. Hence large species are less able to reach high population density, limiting as much their variance in population density (see also Gamelon et al.^70^ for the role of generation time on the population stability of large mammals). The range of the observable values of population density may be further reduced for the larger species due to allometric scaling (sensu constraints) of numerous life history traits, including transient dynamics parameters^70^. On the contrary, the lower spatial variation in population densities of the larger species relative to smaller species might also partly result from their overall poor conservation status, so that high population densities of highly threatened species are not observable. However, as already stated (see above), the spatial variation of population density in large species is higher than expected suggesting rather that our data arose from a mix of particularly high (biased or healthy populations) and low (endangered populations) population densities. Finally, further exploration on the role of other ecological variables potentially impacting rangewide population density (e.g., human footprint index, road density, and net primary productivity), as well as other life traits (i.e., fecundity or dispersal), might identify critical factors impacting felid populations.. For example, the model developed by Segura and Perera^35^ is based on the constraint acting on population abundance through metabolic requirements of species, hence clarifying the role of the local resource availability^33^ on the VMA relationship (i.e. in our case, how the abundance of prey interacts with the VMA relationship).

Such a support for the VMA was obtained by using spatial replicates not conducted at the same spatial scale, for example among our records we observed a gradient of spatial scales, from different parts of a wildlife reserve to national park, through different areas of a country up to by study sites shared among different countries (Supplementary Information Table S1). Despite this, the VMA relationship was supported, albeit we acknowledge that such differences in the spatial scales might also influence the variation observed around the VMA relationship. Moreover, the species with the highest number of spatial replicates were mainly large species (e.g. tiger with 57 spatial replicates), so that the large spatial variation we found in small species cannot be accounted for by a higher number of spatial replicates. The method of density estimation used to record density (CR or SECR estimates) did not alter the slopes of the relationships (i.e. the allometric exponent) as additive models were always the best supported, but rather suggested over- or under-estimation of populations density depending on the methods used (Fig. 2b). During the last years of the study period, most studies used SECR methods to estimate population density and such an approach is becoming the gold standard for estimating the population density in felids. Unfortunately, our data did not allow us to investigate the temporal VMA due to a small number of temporal replicates on a low number of species. However, as the number of camera-trapping studies has increased considerably^71^, it is likely the data required for assessing temporal VMA should be soon available. When temporal replicates were available for a specific study site, we averaged population density over time-period to obtain a single estimate per species of the population density; we acknowledge this approach may have introduced bias as the averaged estimate could not be representative of the true population density for these sites. However, the temporal variance calculated within a single study site was far less important than the spatial variance between sites for these species (results not shown), hence likely minimizing this bias. Finally we used averaged body mass mostly from the study of Johnson et al.^72^ and from the PanTHERIA database; a better approach would have been to obtain the body mass of each species directly from each specific study site. Unfortunately, this information was not available for most study sites. This potential discrepancy between averaged and actual, site-specific body sizes, may have contributed to the variation observed around the VMA.

Our efforts for maintaining the dataset, analyzing it and disseminating our findings^46,47^ have contributed to increase the scientific knowledge for this highly distinctive and charismatic taxon. Moreover, in this study we have found strong support for the VMA law for a whole taxon of carnivore species for the first time and evidently this result calls for other assessments of the VMA at the interspecific level in other taxonomic groups, both on the spatial and temporal variance in population density, before confirming its universality on ecological systems and discussing its implications more in depth.

## Methods

### Data collection

We searched the literature for camera survey-based estimates of population density for felids during 11 January 2012 until 14 December 2019 using the most common, freely accessible and widely used scientific bibliography database: Scirus, Web of Science, BioOne and Google scholar^46,47^. We used the term “camera-trapping” along with both common and scientific names for the 40 species of felids^60^. Each entry was then fully examined and the following data were extracted: species, author (only the first name was recorded), journal name (for technical reports or thesis entries, we used the terms report or thesis as substitute for the journal name), year of publication, study site and specific-study site (for those entries where a specific area within the study site was sampled, see Anile and Devillard^47^ for details), study year(s), sampling dates, the method of density estimation (i.e. either SECR—spatially explicitly capture recapture- or CR—capture recapture-analysis), the type of buffer used (only for CR entries according to the following levels: FMMDM—full mean maximum distance moved; HMMDM—half mean maximum distance moved), and the density *D* (*N/A* individuals/100 km^2^). We retained only these three methods of density estimation as they are recognized to provide the most accurate estimates of the population density of naturally marked individuals from camera trap studies. Until the pioneer study of Efford et al.^73^, researchers have indeed used non-spatial CR models for estimating D; this framework involved the disjointed estimation of N using closed population models and A, the so called “effective” trapping area (i.e. usually a minimum convex polygon delineated around the camera traps plus a buffer). However, this framework underestimates the true movements of animals within and around the trapping grid, hence biasing high the density estimate^59,74^. On the contrary, the development of SECR models overrides the need of estimating the area A because these models inherently estimate D by modelling the location of the home-ranges of the animals (detected and not detected) based on the spatial information provided by the captures themselves^45,75^. We further classified each entry as random *vs*. target records^46,47^ by carefully inspecting the methods section to assess if a species-specific sampling was used for a given species. For entries reporting data for more than one species, year and period (e.g. repeated sampling over the years or repeated sampling in the same year), we considered them independently. Thus, we used only independent records. This strict classification system for each entry was necessary as we found some cases where only the method of density estimation differed or, alternatively, the period of sampling between entries overlapped. In the latter case we only retained the most recent record (i.e. the one with the latest year of publication as in the majority of the cases the SECR method of density estimation was used). For each species we then associated the body mass (mean; gr) using the data provided in Johnson et al.^72^ or alternatively, for those cat species not reported in the above mentioned study, in the PanTHERIA database^76^. The body mass of the Andean cat *Leopardus jacobita* was taken from Huaranca et al.^77^ given it was not reported in the two afore mentioned sources. As *Felidae* are dimorphic species^72^, we averaged male and female body mass.

### Data preparation

From the initial dataset of felid population density estimated trough CR FMMDM, CR HMMDM or SECR method of density estimation, we further reduced the dataset by discarding records from studies where the species was not the targeted one (i.e. random records sensu Anile and Devillard^46^) as relative abundance index (RAI), and hence density estimate, is biased in such studies^46^. A boxplot was used to remove outlier density estimates for each species/method of density estimation pair from the dataset. Particularly high or low density might reveal a bias in the study design and/or peculiar habitat conditions. From this dataset we calculated the mean density *MeanDensity* over specific study sites. When several density estimates were available for a given specific study site (i.e. temporal repeats of the monitoring over years or seasons), we averaged the density estimates over temporal windows. The method of density estimation was accounted for and only specific study sites providing density estimated by the same method were averaged. Therefore a species could have several (up to three) mean densities, one for each method of density estimation. The variance of density *VarDensity* was calculated as the variance of density estimates over specific study sites. Again only the density estimates coming from the same method of density estimation were used to provide a value of *VarDensity* leading potentially to up to three values for *VarDensity* for a single species. For the following data analysis, we only kept in the dataset *MeanDensity* and *VarDensity* that were estimated for at least three different specific study sites.

### Data analysis

We used linear mixed model with a Gaussian distribution with Restricted Maximum Likelihood (REML, *lmer* function in the *lme4* package for the R software) to assess whether the Taylor’s law (TL), the Density-Mass Allometry (DMA) and the Variance-Mass Allometry (VMA) are supported by the density estimates reported in felids from camera-trap studies. Significance threshold was set to nominal value of *α* = 0.05. The continuous response variable was either *VarDensity* (TL, VMA) or *MeanDensity* (DMA), while the explanatory variable was the body mass *BodyMass* for the DMA and VMA tests and the *MeanDensity* in the TL test. For each model (TL, DMA and VMA) we also added as a fixed effect the method of density estimation (*DensityMethod* with three modalities CR FMMDM, CR HMMDM, SECR) in interaction with the continuous explanatory variable. We added *DensityMethod* as a potential confounding factor as previous studies have shown that the method used to estimate density from camera trap studies might affect density estimates^46,59,74,78^. Finally, we included in the models the random factor *Species* because, as stated above, some species can have up to three repeated measures for the pair metric (*MeanDensity*, *VarDensity*) in the dataset coming from the different methods of density estimation.

To ensure that the slope of the TL, DMA and VMA was not biased by the method used to estimate density, we first tested for the interaction between *DensityMethod* and the response variables. If this interaction term was not significant (i.e. the method of density estimation is not affecting the slope), we then computed the additive model to investigate TL, DMA and VMA slope significance, but we kept the method of density estimation in this additive model as a confounding factor. *MeanDensity, VarDensity* and *BodyMass* were log10-transformed in all models. Model residuals were examined for homoscedasticity and normal distribution to assess whether they satisfactorily respected the model hypotheses (Supplementary Information Figs. S2–S4).

## Supplementary information

Supplementary information.

## Data Availability

The datasets generated during and/or analysed during the current study are available from the corresponding author on reasonable request.
