# Author Correction: Spatial variance-mass allometry of population density in felids from camera-trapping studies worldwide

**DOI:** 10.1038/s41598-021-82507-7

**Published:** 2021-01-25

**Authors:** Stefano Anile, Sébastien Devillard

**Affiliations:** 1 Cooperative Wildlife Research Laboratory, Southern Illinois University, Carbondale, IL 62901 USA; 2 Laboratoire de Biométrie Et Biologie Evolutive, Univ Lyon, Université Claude Bernard Lyon 1, CNRS, 69100 Villeurbanne, France

Correction to: *Scientific Reports* https://doi.org/10.1038/s41598-020-71725-0, published online 9 September 2020

The Supplementary Information file that accompanies this Article contains errors in Table S1, where the citation information under columns "First Author", "Publication Year" and "Journal" in row 86 is incorrect.

“Havm√∏ller”, “2018” and “Report”.

should read:

“Havmøller”, “2019” and “PlosOne”.

