# Supplementary material for: Spatial variance-mass allometry of population density in felids from camera-trapping studies worldwide
Source: Sci Rep. 2020 Sep 9;10:14814. doi: 10.1038/s41598-020-71725-0 (PMC7481184; doi:10.1038/s41598-020-71725-0)
Supplement: Supplementary file 1 — Supplementary information. [file 41598_2020_71725_MOESM1_ESM.pdf]

Supplementary information for

Spatial variance-mass allometry of population density in felids from camera-trapping studies worldwide

Stefano Anile[1] & Sébastien Devillard[2*]

[1] Cooperative Wildlife Research Laboratory, Southern Illinois University, Carbondale, IL 620901 USA

[2] Univ Lyon, Université Claude Bernard Lyon 1, CNRS, Laboratoire de Biométrie et Biologie Evolutive, F-69100, Villeurbanne, France.

Table S1 First author, publication year, journal and species of interest for the n=260 articles used to extract data on population density in felids. These 260 articles lead to n=679 records of population density in felids.

First Author	Publication Year	Journal	Species
Alexander	2015	PlosOne	Snow leopard
Alexander	2016	Biological Conservation	Snow leopard
Alexander	2016	Thesis	Puma
Amit	2009	Revista de Ciencias Ambientales (Trop J Environ Sci)	Jaguar
Anile	2010	Italian Journal of Zoology	Wildcat
Anile	2012a	Wildlife Biology in Practice	Wildcat
Anile	2014	Journal of Zoology	Wildcat
Astete Perez	2012	Thesis	Jaguar, Puma
Athreya	2013	PlosOne	Leopard
Avgan	2014	Wildlife Biology	Eurasian lynx
Avila-Najera	2015	International Journal of Tropical Biology and Conservation	Jaguar, Ocelot, Puma
Ayala	2010	Rev. Bol. Ecol. y Cons. Amb	Ocelot
Bahaa-el-din	2016	Biological Conservation	African golden cat
Balme	2010	Animal Conservation	Leopard
Balme	2019	Population Ecology	Leopard
Bashir	2013	Wildlife Biology	Leopard cat
Belbachir	2015	PlosOne	Cheetah
Bisht	2019	Journal of Applied Ecology	Tiger
Blanc	2012	The Journal of Wildlife Management	Eurasian lynx
Boast	2011	Report	Cheetah, Leopard
Bohm	2015	Thesis	Serval
Borah	2012	Report	Clouded leopard, Tiger
Borah	2013	Oryx	Clouded leopard, Leopard

Boron	2016	PlosOne	Jaguar
Braczkowski	2016	PlosOne	Leopard
Brassine	2015	PlosOne	Cheetah
Briers-Louw	2017	Thesis	Leopard
Brodie	2012	Oryx	Sunda clouded leopard
Carrera-Trevino	2016	Revista de Biologia Tropicale	Jaguar
Carter	2012	PNAS	Tiger
Carter	2014	Global Ecology and Conservation	Leopard
Caruso	2012	Annali Zoologici Fennici	Geoffroy's cat, Pampas cat
Chanchani	2014	Report	Tiger
Chapman	2010	South African Journal of Wildlife Research	Leopard
Chase Grey	2013	PlosOne	Leopard
Chauhan	2005	Report	Leopard
Cheyne	2013	Endangered Species Research	Clouded leopard
Chua	2016	Mammal Research	Leopard cat
Clare	2015	The Journal of Wildlife Management	American lynx
Constant	2016	Thesis	Leopard
Cuellar	2006	Studies on Neotropical Fauna and Environment	Geoffroy's cat, Margay, Puma
Das	2012	Report	Tiger
de la Torre	2011	Oryx	Jaguar
de la Torre	2016	Therya	Ocelot
de Thoisy	2016	PlosOne	Jaguar
Devens	2018	African Journal of Ecology	Leopard
Di Bitetti	2006	Journal of Zoology	Ocelot
Diaz-Pulido	2011	Mastozoologia Neotropical	Ocelot
Dorazio	2017	PlosOne	Tiger
du Preez	2014	Biological Conservation	Leopard

Duangchantrasiri	2015	Biological Conservation	Tiger
Edgaonkar	2008	Thesis	Leopard
Edwards	2015	African Journal of Ecology	Leopard
Edwards	2018	African Journal of Ecology	Serval
Espinosa	2018	PlosOne	Jaguar
Faller	2007	Chapter in book	Jaguar
Farhadinia	2019	Scientific Reports	Leopard
Farrington	2016	Report	Snow leopard
Fattebert	2008	Report	Eurasian lynx
Figel	2016	Wildlife Society Bullettin	Jaguar
Fusco-Costa	2010	Mammalian Biology	Ocelot
Gardner	2010	Ecology	Pampas cat
Garrote	2010	European Journal of Wildlife Research	Iberian lynx
Garrote	2012	European Journal of Wildlife Research	Iberian lynx
Gelin	2017	PlosOne	Puma
Ghoddusi	2008	Cat news	Leopard
Gil-Sanchez	2015	Zoology	Wildcat
Gimenez	2019	bioRxiv	Eurasian lynx
Goldberg	2015	PlosOne	Leopard
Gopal	2010	Oryx	Tiger
Goulart	2009	Neotropical Biology and Conservation	Ocelot
Grant	2012	Master Thesis	Leopard
Gray	2012	The Journal of Wildlife Management	Leopard
Grente	2017	Thesis	Jaguar
Guarda	2016	Oryx	Puma
Gubbi	2017	Global Ecology and Conservation	Tiger
Gutierrez-Gonzalez	2012	Oryx	Jaguar

Habib	2014	Report	Leopard
Haines	2006	Oryx	Ocelot
Harihar	2009	Mammalia	Leopard
Harihar	2009	Oryx	Tiger
Harihar	2009	Population Ecology	Tiger
Harihar	2011	Journal of Applied Ecology	Leopard, Tiger
Harihar	2012	PlosOne	Tiger
Harihar	2018	Oryx	Tiger
Havmv∏ller	2018	Report	Leopard
Hearn	2016	PlosOne	Marbled cat
Hearn	2017	Oryx	Sunda clouded leopard
Hedges	2015	Wildlife Society Bullettin	Leopard
Henschel	2008	PhD Thesis	Leopard
Hernandez-Blanco	2013	Doklady Biological Science	Tiger
Hidalgo-Mihart	2019	Hystrix	Jaguar
Huaranca	2019	Wildlife Research	Andean cat, Pampas cat
Isasi-Catala	2013	Chapter in book	Jaguar
Jackson	2005	Report	Snow leopard
Jacques	2019	Wildlife Society Bullettin	American lynx
Janecka	2011	Journal of Mammology	Snow leopard
Jedrzejewski	2016	Mammal Research	Jaguar
Jhala	2011	Report	Tiger
Jimenez	2019	Biological Conservation	Iberian lynx
Johnson	2006	Animal Conservation	Tiger
Kachel	2016	Oryx	Snow leopard
Kalle	2011	Acta Theriologica	Leopard, Tiger
Kane	2015	Biodiversity and Conservation	Lion

Kane	2015	Thesis	Leopard, Serval
Karanth	2004	Animal Conservation	Tiger
Karanth	2005	Report	Tiger
Karki	2011	Thesis	Tiger
Karki	2013	Oryx	Tiger
Kasper	2015	Iheringia Serie Zoologia	Ocelot
Kawanishi	2004	Biological Conservation	Tiger
Kent	2011	PhD Thesis	Leopard
Khan	2012	Journal of Threatened Taxa	Tiger
Kilshaw	2011	Report	Wildcat
Kolowski	2010	Biological Conservation	Ocelot
Kubala	2017	Oryx	Eurasian lynx
Kumar	2019	Scientific Reports	Leopard, Tiger
Kunz	2017	Report	Eurasian lynx
Kutal	2015	Chapter in book	Eurasian lynx
Lamicchane	2017	Oryx	Tiger
Lamicchane	2019	Biodiversity and Conservation	Leopard
Larrucea	2007	Western North American Naturalist	American lynx
Lham	2016	Report	Snow leopard
Lingaraja	2017	Current Science	Tiger
Linkie	2006	Journal of Applied Ecology	Tiger
Linkie	2008	Biological Conservation	Tiger
Loken	2016	PhD Thesis	Sunda clouded leopard
Lombardi	2017	Urban Ecosystem	American lynx
Loock	2018	bioRxiv	Serval
Luskin	2017	Nature communications	Tiger
Lynam	2009	Population Ecology	Tiger

Maffei	2005	Journal of Tropical Ecology	Jaguar, Ocelot
Majumder	2011	Thesis	Leopard, Tiger
Majumder	2017	European Journal of Wildlife Research	Tiger
Marker	2008	Cat news	Cheetah
Martinez-Hernandez	2015	Oryx	Ocelot
Massara	2015	PlosOne	Ocelot
Mazzolli	2010	Environmental Management	Puma
Medellin	2016	Report	Jaguar
Miquelle	2012	Report	Leopard
Mohamed	2013	Journal of Mammalogy	Leopard cat
Mohamed	2019	Oryx	Sunda clouded leopard
Mondal	2012	International Journal of Biodiversity and Conservation	Leopard
Mondal	2012	World Journal of Zoology	Leopard
Monterrubio-Rico	2017	Mammalia	Ocelot
Moreira	2008	Mesoamerican	Puma
Moreno	2009	Tecnociencia	Ocelot
Mosquera	2016	Neotropical Biodiversity	Ocelot
Mrozinski	2018	Thesis	Puma
Murphy	2019	Scientific Reports	Puma
Naing	2017	Oryx	Clouded leopard, Marbled cat, Tiger
Negroes	2010	Journal of Wildlife Management	Puma
Negroes	2012	Ecological Research	Jaguar
Ngoprasert	2019	Mammalian Biology	Tiger
Noack	2019	Animals	Leopard
Noss	2012	Animal Conservation	Jaguar, Ocelot, Puma
Nunez-Perez	2011	Journal of Zoology	Jaguar

O'Brien	2003	Animal Conservation	Tiger
O'Brien	2011	Ecology	Cheetah, Leopard
Palom-Munoz	2015	Thesis	Ocelot
Paviolo	2008	Oryx	Jaguar
Paviolo	2016	Scientific Reports	Jaguar
Penido	2016	Biota Neotropical	Ocelot
Penjor	2018	Ecology and Evolution	Clouded leopard
Pereira	2011	Journal of Zoology	Geoffroy's cat
Perez-Irineo	2014	Inicio	Ocelot
Perez-Irineo	2017	Therya	Margay, Ocelot
Pesenti	2013	Journal of Mammalogy	Eurasian lynx
Petersen	2019	Journal of Threatened Taxa	Leopard cat
Petit	2017	Mammalia	Jaguar
Pokheral Chiranjibi	2011	Thesis	Leopard, Tiger
Pusparini	2017	Oryx	Tiger
Qi	2015	Biological Conservation	Leopard
Quiroga	2016	Journal for Nature Conservation	Puma
Rahman	2018	Folia Zoologica	Leopard
Ramalho	2012	Thesis	Jaguar
Ramesh	2012	Tropical Ecology	Leopard, Tiger
Ramesh	2013	Journal of Mammalogy	Serval
Ramesh	2017	Ecology and Evolution	Leopard
Ray	2011	Thesis	Leopard
Rayan	2009	Oryx	Tiger
Rayan	2015	Biological Conservation	Tiger
Razak	2019	IOP Conf. Series: Earth and Environmental Science	Clouded leopard
Reppucci	2011	Journal of Mammalogy	Andean cat

Rich	2014	Journal of Mammology	Puma
Rich	2019	Biological Conservation	Leopard, Lion, Serval
Rocha	2016	PlosOne	Ocelot
Rodgers	2014	Tropical Conservation Science	Ocelot
Rostro-Garcia	2018	Royal Society Open Science	Leopard
Roy	2015	Population Ecology	Tiger
Royle	2009	Ecology	Tiger
Sadhu	2017	BMC Zoology	Tiger
Salom-Perez	2007	Oryx	Jaguar
Salvador	2015	Mammalia	Ocelot
Sankar	2012	Report	Tiger
Sarmento	2019	Mammalian Biology	Iberian lynx
Satter	2017	Thesis	Ocelot
Scott Eaton	2016	Thesis	Jaguar
Selvan	2014	Mammalia	Leopard cat
Selvan	2014	Mammalian Biology	Leopard, Tiger
Sharma	2011	Population Ecology	Tiger
Silveira	2009	Oryx	Jaguar
Silver	2004	Oryx	Jaguar
Simcharoen	2007	Oryx	Tiger
Singh	2014	SpringerPlus	Tiger
Singh	2014	Wildlife Society Bullettin	Tiger
Singh	2017	Journal of Mammalogy	Clouded leopard, Marbled cat
Smolko	2018	Report	Eurasian lynx
Soisalo	2006	Biological Conservation	Jaguar
Sollmann	2011	Biological Conservation	Jaguar
Sollmann	2013	Journal of Applied Ecology	Puma

Sollmann	2014	Oryx	Sunda clouded leopard
Soria-Diaz	2010	Animal Biology	Puma
Soutyrina	2013	Report	Tiger
Srbek-Araujo	2016	Oryx	Jaguar
Srivatasha	2015	Journal of Mammalogy	Leopard cat
Strampelli	2015	Thesis	Leopard
Sunarto	2013	Oryx	Tiger
Swanepoel	2015	Wildlife Biology	Leopard
Thapa	2014	Advances in Ecology	Leopard
Thapa	2016	Integrative Zoology	Leopard, Tiger
Thempa	2016	Thesis	Tiger
Thiel	2011	Thesis	Serval
Thinley	2014	Report	Snow leopard
Thinley	2015	Bhutan Journal of Natural Resources & Development	Tiger
Thornton	2015	Wildlife Research	American lynx
Tirelli	2019	Mammalian Biology	Geoffroy's cat
Tobler	2013	Biological Conservation	Jaguar
Tobler	2018	Biological Conservation	Jaguar
Trinca	2014	Thesis	Jaguar, Ocelot
Trolle	2003	Journal of Mammology	Ocelot
Trolle	2005	Mammalia	Ocelot
Valderrama-Vasquez	2013	Chapter in book	Ocelot
Vitkalova	2018	Conservation Letters	Leopard
Wan Mohamad	2015	Population Ecology	Clouded leopard
Wang	2009	Biological Conservation	Leopard, Tiger
Wang	2016	Biological Conservation	Leopard

Wegge	2009	Biological Conservation	Tiger
Wegge	2016	Oryx	Tiger
Weingarth	2012	Animal Biodiversity and Conservation	Eurasian lynx
Wibisono	2009	Oryx	Tiger
Williams	2017	Royal Society Open Science	Leopard
Wilting	2012	Oryx	Sunda clouded leopard
Wolff	2019	Hystrix	Ocelot
Wultsch	2013	Thesis	Jaguar
Xiao	2016	Integrative Zoology	Tiger
Young	2019	Urban Ecosystem	American lynx
Zanon-Martinez	2016	Wildlife Research	Puma
Zimmerman	2007	Report	Eurasian lynx
Zimmerman	2009	Report	Eurasian lynx
Zimmerman	2011	Report	Eurasian lynx
Zimmerman	2012a	Report	Eurasian lynx
Zimmerman	2012b	Report	Eurasian lynx
Zimmerman	2013	Integrative Zoology	Eurasian lynx
Zimmerman	2015a	Report	Eurasian lynx
Zimmerman	2015b	Report	Eurasian lynx
Zimmerman	2016a	Chapter in book	Eurasian lynx
Zimmerman	2016b	Report	Eurasian lynx
Zimmerman	2017	Report	Eurasian lynx
Zwicker	2015	Thesis	Jaguar

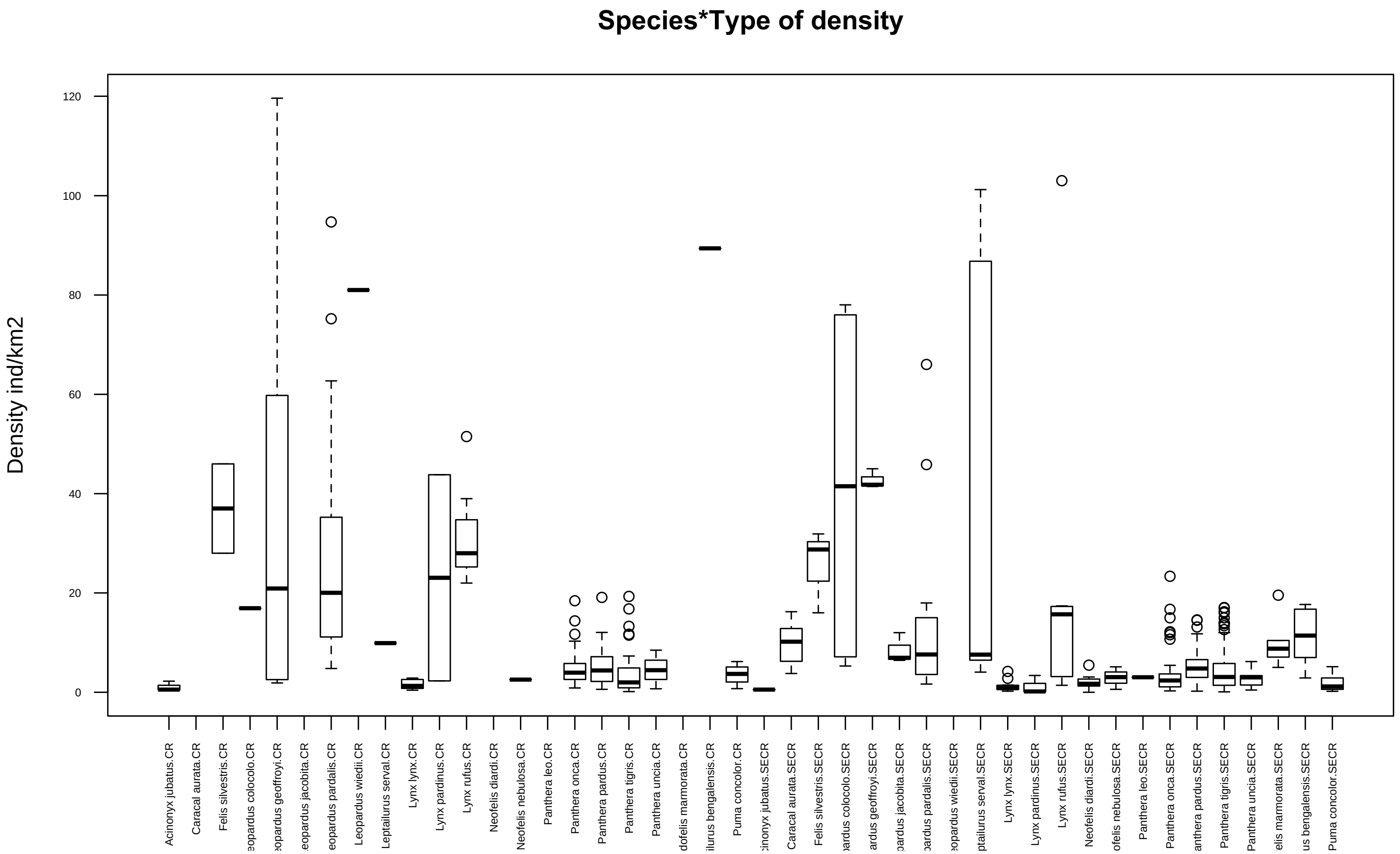

Figure S1 Boxplot of the n=589 records (i.e. starting dataset of 679 records minus n=90 random surveys, see methods) showing the discarded outliers (n=38) from the dataset.

Table S2 Mean density (*MeanDensity ind/km2*), variance of density estimates (*VarDensity*) over different study sites and body mass (*kg*) for the n=22 documented felids species. The number of spatial replicates per species/type of density pair is also shown. These data were subsequently used for modelling Taylor's Law (TL), Density-Mass Allometry (DMA) and Variance-Mass Allometry (VMA).

**Specie**	**Scientific name**	**Type of Density**	**Mean Body Mass**	**Mean density *MeanDensity***	**Variance in density *VarDensity***	**# study sites**
Ocelot	*Leopardus pardalis*	CR FMMDM	11.65	19.08	166.56	4
Tiger	*Panthera tigris*	CR FMMDM	190.50	2.22	1.97	7
Geoffroy's cat	*Leopardus geoffroyi*	CR HMMDM	4.65	30.56	1400.83	6
Ocelot	*Leopardus pardalis*	CR HMMDM	11.65	28,83	490.58	15
Eurasian lynx	*Lynx lynx*	CR HMMDM	18.45	1.49	0.52	9
Bobcat lynx	*Lynx rufus*	CR HMMDM	7.75	28.33	38.57	6
Jaguar	*Panthera onca*	CR HMMDM	85.70	4.04	4.50	22
Leopard	*Panthera pardus*	CR HMMDM	41.00	4.80	12.44	15
Tiger	*Panthera tigris*	CR HMMDM	190.50	2.66	4.21	18
Puma	*Puma concolor*	CR HMMDM	44.80	3.45	7.43	3
African golden cat	*Caracal aurata*	SECR	9.10	9.86	24.83	5
Wildcat	*Felis silvestris*	SECR	4.30	25.55	70.88	3
pampas cat	*Leopardus colocolo*	SECR	3.95	30.10	1583.49	3
Andean cat	*Leopardus jacobita*	SECR	5.16	8.09	6.85	4
Ocelot	*Leopardus pardalis*	SECR	11.65	8.16	22.22	6
Serval	*Leptailurus serval*	SECR	9.85	23.25	1461.62	5
Eurasian lynx	*Lynx lynx*	SECR	18.45	0.76	0.14	9
Bobcat lynx	*Lynx rufus*	SECR	7.75	9.67	61.33	6
Sunda clouded leopard	*Neofelis diardi*	SECR	19.90	1.82	0.87	11
Clouded leopard	*Neofelis nebulosa*	SECR	17.00	2.93	5.16	3
Jaguar	*Panthera onca*	SECR	85.70	2.04	1.75	42

Leopard	*Panthera pardus*	SECR	41.00	4.09	6.76	48
Tiger	*Panthera tigris*	SECR	190.50	3.44	9.23	57
Snow leopard	*Panthera uncia*	SECR	40.25	3.18	5.25	7
Marbled cat	*Pardofelis marmorata*	SECR	2.83	7.85	5.39	4
Leopard cat	*Prionailurus bengalensis*	SECR	2.60	11.37	31.79	8
Puma	*Puma concolor*	SECR	44.80	1.47	2.17	15

Diagnostic plots for the goodness of fit of linear mixed models used to investigate TL, DMA and VMA

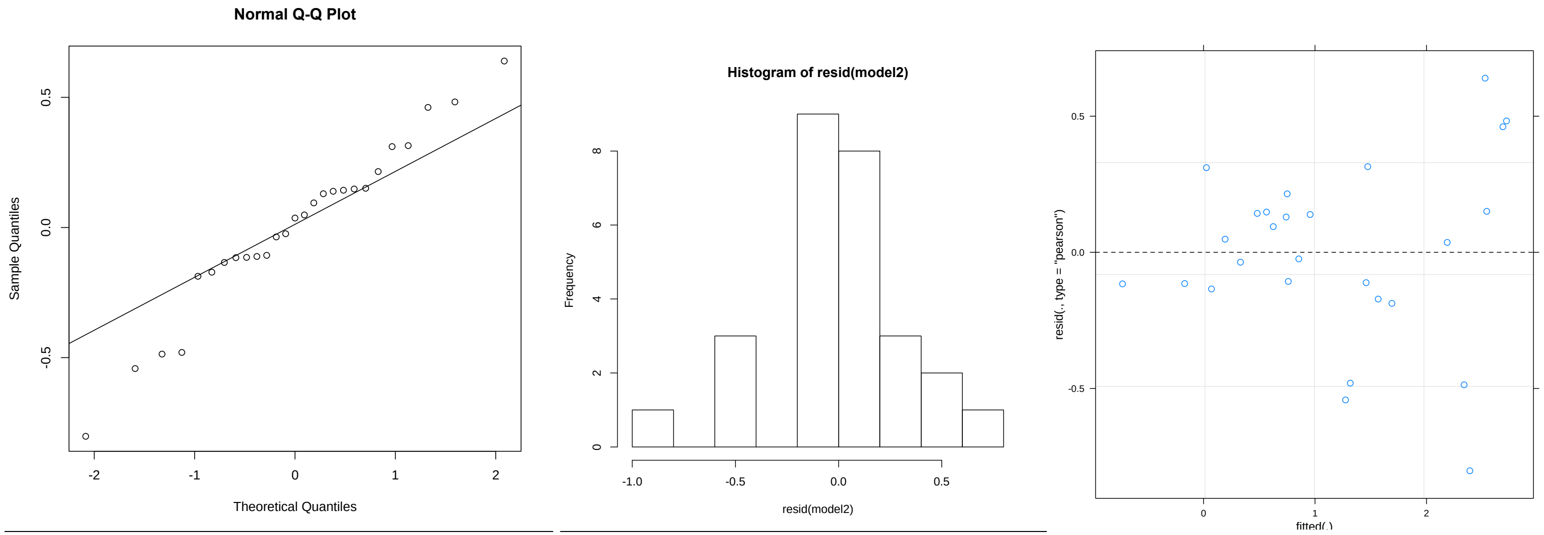

Figure S2 QQ plot of model residuals (Normality assumption), histogramm of model residuals (Normality assumption) and standardized residuals vs. fitted residuals plot (Homoscedacticity assumption) for the linear mixed model testing the Taylor’s law on Felids.

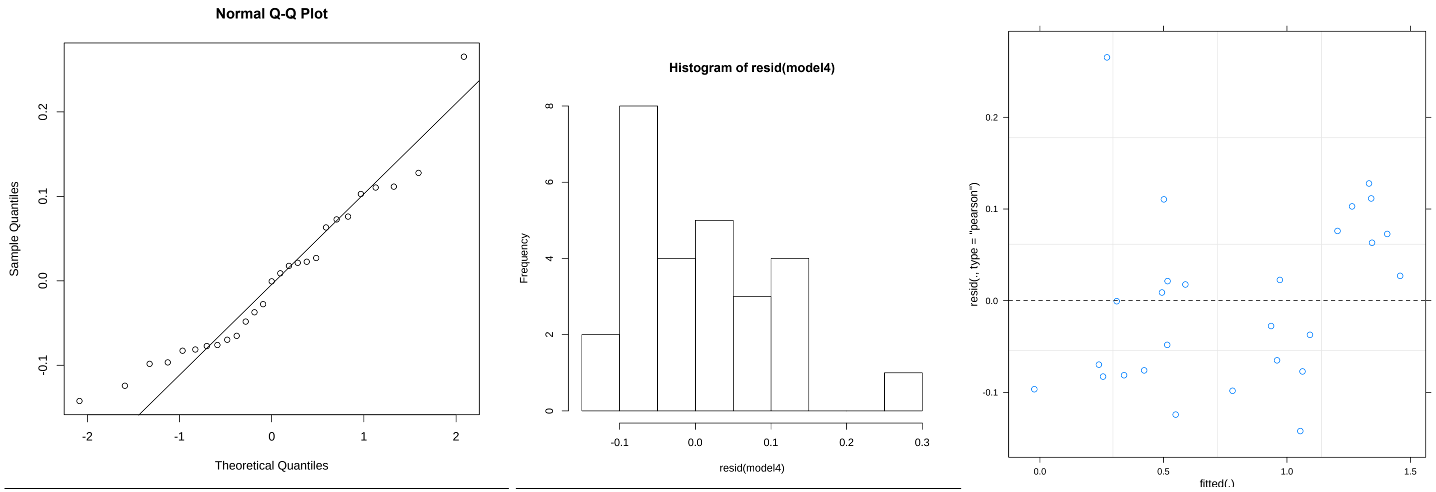

Figure S3 QQ plot of model residuals (Normality assumption), histogramm of model residuals (Normality assumption) and standardized residuals vs. fitted residuals plot (Homoscedacticity assumption) for the linear mixed model testing the Density-Mass allometry on Felids.

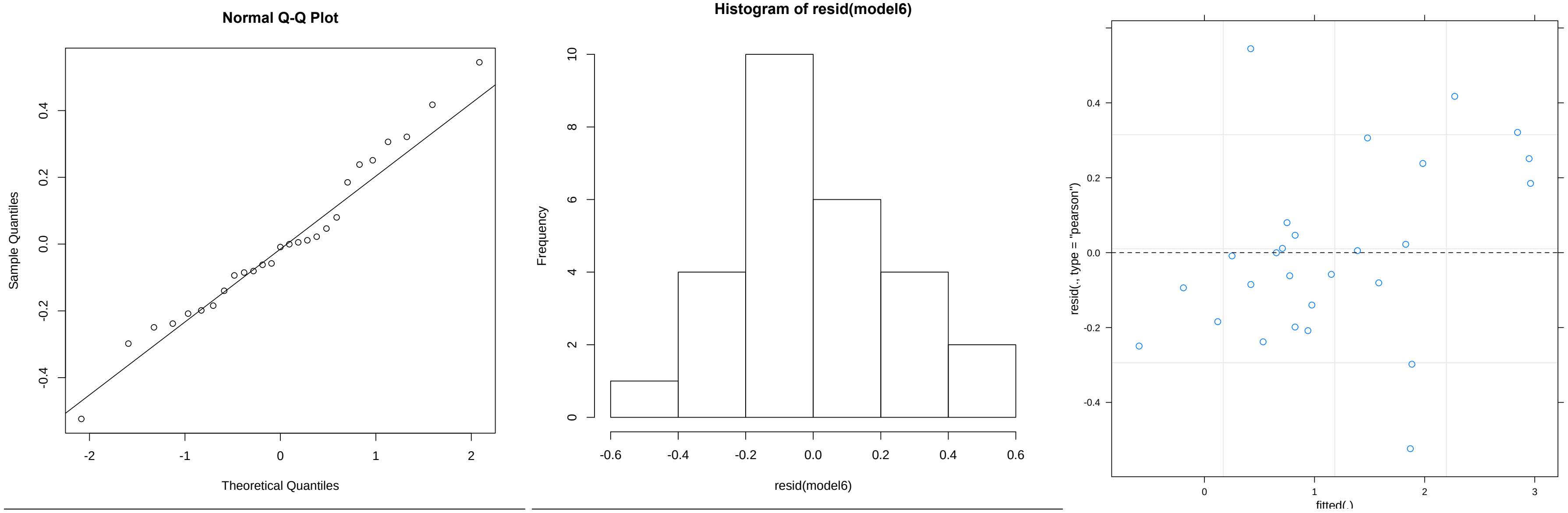

Figure S4 QQ plot of model residuals (Normality assumption), histogramm of model residuals (Normality assumption) and standardized residuals vs. fitted residuals plot (Homoscedacticity assumption) for the linear mixed model testing the Variance-Mass allometry on Felids.
